# Determinants of Preterm Birth among Mothers Who Gave Birth in Dilla University Referral Hospital, Southern Ethiopia: A Case-Control Study

**DOI:** 10.1155/2020/7031093

**Published:** 2020-12-15

**Authors:** Dagmawit Wakeyo, Yohannes Addisu, Moges Mareg

**Affiliations:** ^1^Department of Reproductive Health, School of Public Health, College of Health Science and Medicine, Dilla University, Dilla, Ethiopia; ^2^School of Public Health, College of Health Science and Medicine, Dilla University, Dilla, Ethiopia

## Abstract

Globally, every year, 1.1 million newborns die due to prematurity. In Ethiopia, 320,000 preterm births occur each year; out of these, 24,400 deaths were due to preterm complications. However, there is little evidence about preterm birth in the study area. Therefore, this study provides an important direction for health professionals, health programmers, and researchers. A facility-based unmatched case-control study design was employed among 244 women (61 cases and 183 controls) who gave birth in Dilla University Referral Hospital and were selected with purposive sampling. The bivariate and multivariable logistic regression model was used to select independent predictors of preterm birth. The multivariate analysis was used, and the results were interpreted using an adjusted odds ratio at 95% confidence interval and statistically significant level at a *P* value less than 0.05. A total of 240 mothers (60 cases and 180 controls) were included in the study with a 98.3% response rate. Factors like attending secondary educational and above [adjusted odd ratio (aOR) = 0.07 (0.08-0.65)] and attending antenatal care [aOR = 0.41 (0.18-0.93)] were protective whereas having urinary tract infection [aOR = 3.6 (1.1-11)], having human immune virus diseases [aOR = 4.2 (0.9-18)], having a history of abortion [aOR = 2.3 (1.1-5)], having a history of preterm delivery [aOR = 5 (1.6-15)], and having hypertensive disorders of pregnancy [aOR = 5 (1.9-13)] were significantly associated risk factors for preterm birth. The main determinant factors for preterm birth are having antenatal care follow-up, attending secondary education and above, hypertensive disorders of pregnancy, having HIV/AIDS, and history of abortion. This shows a need to strengthen female education; screen mothers for HIV/AIDS, urinary tract infection, and hypertension; and strengthen nutritional counseling, during ANC visits.

## 1. Introduction

Preterm birth is defined as all births before 37 completed weeks of gestation or less than 259 days since the first day of a woman's last normal menstrual period. It is classified as medically indicated (iatrogenic) and spontaneous (idiopathic) preterm birth. And according to gestational age, it is also classified as extremely preterm (<28 weeks), very preterm (28 to <32 weeks), and moderate to late preterm (32 to <37 weeks). It is the leading cause of prenatal morbidity and mortality in both developed and developing countries including Ethiopia. In most cases, preterm deliveries occur spontaneously and are associated with long-term complications in surviving infants [1].

The majority of global preterm births occur in Asia and Africa where health systems are weak and access to utilization of health services is limited [2]. The lowest rate of preterm birth occurred in Europe (6.2%) and the highest rates occurred in Africa (11.9%) and North America (10.6%) [3–5]. According to different studies in Ethiopia, the prevalence of preterm birth is 4.4% in Gondar [6], 16.5% in Addis Ababa [7], and 25.9% in Jimma [8], whereas there is limited evidence in the study area especially related to the determinant factors of preterm birth.

The global community committed to the Sustainable Development Goals and Every Woman and Every Child initiatives to prevent and improve survival of preterm births; different interventions have been implemented which need huge financial capacity [9]. Some of the interventions include antenatal corticosteroid, antibiotic, kangaroo mother care, immediate intensive care unit service, and long-term complex health needs [2, 5]. These interventions lead to a high economic burden for the family and the community at large through implementations of programs, policies, and strategies [8].

The national-level profile provides the most current national-level information on the status of prevention and cares for preterm birth and low birth weight in Ethiopia. More effort is needed to identify women at risk of preterm labor and support them to give birth in a health facility that can offer extra care when needed, such as support for adequate feeding with breast milk, continuous skin to skin contact, antibiotics, and antenatal corticosteroids [2]. To do this, families, communities, and health care workers must value small babies so that they receive the life-saving care they need [5].

The preterm birth causes significant health consequences to the infant and economic costs for families and communities. In advances, prenatal and neonatal care have improved the survival of preterm infants, but those infants who do survive have a risk of developmental disabilities, health and growth problems, chronic diseases, and mortality later in life [1] than infants born at >37 weeks of gestation [10]. About 75% of prenatal deaths and 50% of neurological abnormalities are directly related to preterm birth [11]. The increased prevalence of medical disabilities, learning difficulties, and behavioral and psychological problems among surviving preterm infants has raised concerns that these infants may have difficulties in coping during the stage of adult life [12].

According to the Gedeo Zone maternal and child health (MCH) report in 2017, preterm births were 122 (0.6%); from these, 103 (84%) were alive and 19 (16%) were dead (Gedeo Zone health department report, 2017). This study is aimed at identifying the determinants factors of preterm birth in the Gedeo Zone, Dilla University Referral Hospital.

## 2. Methods

### 2.1. Study Design and Setting

A facility-based unmatched case-control study was employed from May 5^th^ to July 18^th^, 2018. The study was conducted at Dilla University Referral Hospital which is situated in Dilla town, Gedeo Zone, South Ethiopia. Dilla town is located 360 km away from Addis Ababa, the capital city of Ethiopia. This referral hospital has seven departments: surgical, gynecologic/obstetric, medical, radiology, dermatology, psychiatry, and pediatric wards. Taking into account the better curative services provided for, it is one of the major important destinations for patients coming from all directions of the zone, including other bounding regions like the Oromia region and Sidama and Amaro Kelle zones [13].

### 2.2. Population and Participants

Women who gave birth less than 37 weeks of gestation were cases, and women who gave birth greater than or equal to 37 weeks of gestation were controls. The gestational age was calculated based on the last normal menstrual period (LNMP) or ultrasound examination (for those who have early ultrasound examination). All the cases and controls were recruited during the time of child birth.

The sample size was computed using a formula for two population proportions and calculated by OpenEpi version 3.03 statistical software package by considering 23.6% of cases and 7.5% of controls exposed taken from a study done in Addis Ababa public hospitals [7]. After adjustment for 10% nonresponse rate, the final sample size for cases was 61 and for controls was 183. Finally, the total sample size becomes 244. Purposive sampling technique was used to select the study participants. Seven hundred twenty-nine mothers who gave birth in Dilla University Referral Hospital during the study period were the study population. Mothers who had serious illness (unable to communicate during data collection) and edema, which may result in defect on mid-upper arm circumference (MUAC) measurement, were excluded.

### 2.3. Variables of the Study

The outcome of the study was preterm birth, and the independent variables were sociodemographic characteristics (maternal age, marital status, family size, educational status, residence, occupational status, and family income), obstetrics-related variables (antenatal follow-up, history of abortion, history of still birth, history of bleeding, and medication use during pregnancy), medical condition (hypertension, diabetes mellitus, HIV/AIDS, and urinary tract infection), and maternal nutritional factor (mid-upper arm circumference (MUAC)).

### 2.4. Data Collection Instrument

Data collection instruments were adapted from the Ethiopia Demographic and Health Survey (EDHS) and different kinds of literature [14–17]. It is composed of sociodemographic factors, obstetric, medical condition, and maternal nutritional factors.

The outcome variable preterm birth and independent variables like sociodemographic, obstetric, and medical history were collected from the mother by using a structured questionnaire by direct interview, and data on medical factors were extracted from medical records. Gestational age was measured by clinicians either through the last normal menstrual period (LNMP) or ultrasound examination of those women who had preterm birth.

Mid-upper arm circumference (MUAC) was measured by using flexible nonstretchable tape taken at the midpoint of the left upper arm at the relaxed position without any clothing and with optimal tape tension between the acromion process on the shoulder blade and tip of olecranon process of the ulna.

The questionnaire was prepared in English and translated to the local language and then backtranslated to English to check the consistency. Data collection was done by trained diploma nurses. Two-day training was given for data collectors on the objectives of the study, the contents of the questionnaire, issues related to the confidentiality of the responses, and the rights of the respondents. Pretest was done at the Yirgacheffe District Hospital on 10% (24 women), which is 24 km away from Dilla town. The collected data was checked daily by the principal investigator, supervisors, and data collectors to reduce incomplete data.

### 2.5. Data Processing, Analysis, and Presentation

First, the data were checked for their completeness and consistency and entered into OpenEpi version 3.03 exported to SPSS statistical software version 20 for analysis. Descriptive statistics were used to measure the characteristics of the study participants. Both bivariable and multivariable logistic regression analysis were computed to identify factors associated with preterm birth. Variables with a *P* value of less than 0.25 in a bivariable analysis were considered as candidates for multivariable logistic regression. In multivariable analysis, a variable with a *P* value of ≤0.05 was considered as having statistically significant association with preterm birth. The level of the association was measured by using an odds ratio (OR) with corresponding 95% confidence interval (CI).

### 2.6. Ethical Approval and Consent to Participate

The study was approved by the institutional review board (IRB) of Dilla University College of Health Science and Medicine. For the purpose of data collection from the participants, an explanation was given to them regarding to the purpose of the study, the importance of their participation, and true responses. It was also explained that the study had no connection with the individual affairs of respondents. All sampled populations were encouraged to participate in the study, while at the same time, their right not to participate was also respected. Volunteer participants signed written informed consent. For those participants less than 18 years old, we got written consent from their parents and guardians and assent from them. Women who have a preterm baby in the data collection period were counseled about nutrition and provision of care to their child.

## 3. Results

From a total of 244 women, 240 (60 cases and 180 controls) responded, and the response rate was 98.3% in both cases and controls. The case and control groups have comparable sociodemographic characteristics. The mean maternal age in both groups was 29 (SD ± 5.4) years. In both study groups, more than 86.6% of the women were married. Among cases, 6 (10%) attended tertiary education while the proportion of mothers who attended above tertiary education among controls was 56 (31%) (Table 1).

### 3.1. Maternal Obstetric and Medical Characteristics

Regarding the ANC service utilization during pregnancies, 35 (58%) of the cases and 145 (81%) of the controls had ANC follow-up. Twenty-three percent of the cases and 31% of the controls had 4 or more ANC visits during pregnancy. Around 27% of the cases and 11% of the controls had hypertensive disorders of pregnancy (HDP). About 11 (18%) of the cases and 8 (4%) of the controls have a history of preterm birth. Nine (15%) of the cases and 5 (2.7%) of the controls were HIV positive. Thirty-eight percent of the cases and 23.3% of the controls had a history of abortion in the previous pregnancy. Regarding medication use, 20% of the cases and 14.4% of the controls use medication during pregnancy. Fifty-eight percent of the cases and 52.8% of the controls had more than 5 family sizes (Table 2).

### 3.2. Determinants of Preterm Birth

Variables that have a *P* value of less than 0.25 were moved to multivariable logistic regression analysis to determine the predictors of preterm birth. Based on the final multivariable analysis model, variables that were identified as independent predictors of preterm birth were as follows: attending education secondary and above level, having ANC follow-up, HIV-positive serostatus, having urinary tract infection, history of abortion, history of preterm birth, and hypertensive disorder of pregnancy.

In this finding, women who had attended tertiary education were 93% less likely to have preterm birth as compared to those who had not attended education (OR = 0.07, CI = 0.08 − 0.65). Women who had ANC follow-up were 59% less likely to have preterm birth as compared to those who had no ANC follow-up (OR = 0.41, CI = 0.18 − 0.93]. Women who had hypertension disorder during pregnancy were 5 times more likely to have preterm birth as compared to those who had no hypertension disorder during pregnancy (OR = 5, CI = 1.9 − 13). Women who had urinary tract infection during pregnancy were 3.6 times more likely to have preterm birth as compared to those who have no urinary tract infection during pregnancy (OR = 3.6, CI = 1.1 − 11). HIV-positive women were 4.2 times more likely to have a preterm baby as compared to their counterparts (OR = 4.2, CI = 0.9 − 18). Those women who had a history of preterm birth were 5 times more likely to have preterm birth as compared to their counterparts (OR = 5, CI = 1.6 − 15). Women who had a history of abortion were 2.3 times more likely to have preterm birth as compared to those who had no history of abortion (OR = 2.3, CI = 1.1 − 5) (Table 3).

## 4. Discussion

This study was an unmatched case-control study on 240 mothers who gave birth at Dilla University Referral Hospital intending to identify the determinant of preterm births. According to this study, the variables like educational status, receiving antenatal care, having hypertensive disorders of pregnancy, HIV/AIDS-positive serostatus, having urinary tract infection, having a history of preterm birth, and history of abortion have a statistically significant association with preterm birth.

Women who had attended tertiary and above education were 93% less likely to have preterm birth as compared to those who cannot read and write. This finding is in agreement with a study done in Jimma [8]; Bengal, India [18]; and Bangladesh [19]; women who had tertiary and above education had a low risk of preterm delivery as compared to their counterparts. This might be because when a woman becomes more educated, she will have better access to information, knowledge on different health problems, and prevention skills.

The other factor found to be associated with preterm birth in this study was ANC visit. Mothers who had ANC visit had a 59% decreased risk of preterm birth than mothers who had no ANC visit. This finding is supported by a study done in Debre Markos [20]; Bengal, India [18]; Tanzania [21]; Brazil [22]; and Bangladesh [19]. This might be because when the mothers have an ANC visit, they may be counseled by health professionals about early identification of risk factors, signs and symptoms, diagnosis, and treatment of preterm births in the health facilities.

This study revealed that women who had urinary tract infections during pregnancy were 3.6 times more likely to have preterm birth as compared to those who had no urinary tract infection during pregnancy. This result agreed with the study done in Tanzania [21] and Kenya [23]. Urinary tract infections can weaken the membranes of the amniotic sac around the baby. This could lead to premature rupture of the membranes and preterm labor [14].

The history of abortion is another significant risk factor associated with preterm birth. Women who had a history of abortion were 2.3 times more likely to have preterm birth as compared to those who had no history of abortion. This finding is similar to the studies conducted in Jimma [8] and Kenya [23]. The fact that abortion increases the risk of preterm birth is that surgical evacuation of the uterus mechanically stretches the cervix which predisposes such mothers to preterm birth in consecutive pregnancies [24].

Women who had a history of preterm birth were 5 times more likely to have preterm birth as compared to those who had no history of preterm birth. This finding agreed with studies done in Jimma [8]; Western Maharashtra [25]; Bengal, India [18]; and Kenya [23]. The mechanism for this has not been well understood; however, the likelihood of such experience among the women with prior spontaneous labor as well as those with inducing preterm birth is rising [10].

Another determinant of preterm birth is hypertension during pregnancy. Women who had hypertension during pregnancy were 5 times more likely to have preterm birth as compared to those who had no hypertension during pregnancy. This study is in agreement with the study done in Gondar [6], Ghana [26], Kenya [23], and Nigeria [11]. This might be because complications of pregnancy-induced hypertension can cause vascular damage to the placenta or iatrogenesis due to the severity of hypertension or its complications. This in turn induces the oxytocin receptors, which results in preterm labor and delivery, [12].

Being HIV/AIDS positive is also significantly associated with preterm birth. Women who are HIV positive were 4.2 times more likely to have preterm birth as compared to their counterparts. This finding is similar to studies done in Addis Ababa [7] and Tanzania [21] where women with infection during pregnancy were more likely to deliver a preterm baby than their counterparts. This might be due to the effect of antiretroviral drugs, and the reduced immunity of women is a risk factor for preterm birth [11].

Finally the limitation of the study was recall bias, being a case control study; it can only tell us the presence of association but not causal relationship. To alleviate such recall biases, the data collectors cross-checked the data from medical records of the participants.

## 5. Conclusion

This study showed the determinant factors like education level secondary and above and having antenatal care visit were protective factors, whereas having hypertension during pregnancy, HIV/AIDS-positive serostatus, having urinary tract infection, history of preterm birth, and history of abortion were risk factors for preterm birth. This shows a need for strengthening of female education; early screening and treatment of maternal HIV/AIDS, urinary tract infection, and hypertension; strengthening of antenatal care follow-up; nutritional counseling during ANC visit; and prevention of the occurrence of abortion is recommended to reduce the preterm birth.

## Figures and Tables

**Table 1:** Sociodemographic characteristics of women who gave birth at Dilla University Referral Hospital, Gedeo Zone, Southern Ethiopia, May 5–July 18, 2018 (*n* = 240).

Variable	Study subjects	
	Case (*n*, %) *n* = 60	Control (*n*, %) *n* = 180
Age		
15-24	14 (23)	29 (16)
25-34	39 (65)	108 (60)
≥35	7 (12)	43 (24)

Ethnicity		
Gedeo	32 (53.3)	74 (41)
Oromo	11 (18.3)	37 (20.5)
Amhara	3 (5)	19 (10.5)
Other^a^	14 (23.4)	50 (28)

Marital status		
Married	52 (86.6)	174 (96.6)
Divorce and widowed	8 (13.4)	6 (3.4)

Educational status of the mother		
Cannot read and write	36 (60)	53 (29)
Primary education	6 (10)	12 (7)
Secondary education	12 (20)	59 (33)
Tertiary and above	6 (10)	56 (31)

Residences		
Urban	29 (48.3)	77 (42.7)
Rural	31 (51.7)	103 (57.2)

Monthly income (Ethiopian birr)		
<600 ETB	25 (42)	59 (33)
≥600 ETB	35 (58)	121 (67)

Farmer, daily laborer, student. ^a^Sidama, Wolita, and Gurage.

**Table 2:** Obstetric and medical characteristics of women who gave birth at Dilla University Referral Hospital, Gedeo Zone, Southern Ethiopia, May 5–July 18, 2018 (*n* = 240).

Variable	Study subjects	
	Cases (number %)	Controls (number %)
Attending antenatal care (ANC)		
Yes	35 (58)	145 (81)
No	25 (42)	35 (19)

Number of ANC visits		
1 times	11 (18)	22 (12)
2-3times	10 (16)	67 (37)
≥4 times	14 (23)	56 (31)

Family size		
1-2	1 (2)	2 (1.2)
3-4	24 (40)	83 (46)
≥5	35 (58)	95 (52.8)

Hypertensive disorders of pregnancy		
Yes	16 (27)	20 (11)
No	44 (73)	160 (89)

UTI (urinary tract infection)		
Yes	11 (18.3)	13 (7.2)
No	49 (81.7)	167 (92.8)

HIV/AIDS serostatus		
Positives	9 (15)	5 (2.7)
Not positive	51 (85)	175 (97.3)

History of preterm birth		
Yes	11 (18)	8 (4)
No	49 (82)	172 (96)

History of abortion		
Yes	23 (38)	42 (23.3)
No	37 (62)	138 (76.7)

Medication use during pregnancy		
Yes	10 (20)	26 (14.4)
No	50 (80)	154 (85.5)

**Table 3:** Multivariate analysis for determinants of preterm birth among mothers who gave birth at Dilla University Referral Hospital, Southern Ethiopia, May 5–July 18, 2018 (*n* = 240).

Variable	Study subjects		COR (95% CI)	AOR (95% CI)
	Cases (*n*, %)	Controls (*n*, %)		
Educational status of mother				
Illiterate	36 (60)	53 (29)	1	1
Primary education	6 (10)	12 (7)	0.73 (0.25-2.14)	0.59 (0.16-2.1)
Secondary education	12 (20)	59 (33)	0.29 (0.14-0.63)	0.2 (0.07-0.53)^∗∗^
Tertiary and above	6 (10)	56 (31)	0.16 (0.06-0.4)	0.07 (0.08-0.65)^∗∗^

Occupation of the mother				
Housewife	35 (58)	60 (33)	1	1
Merchant	13 (22)	57 (32)	0.39 (0.18-0.81)	0.8 (0.32-1.7)
Employee	6 (10)	48 (27)	0.21 (0.083-0.55)	3.2 (0.35-30)
Other^∗^	6 (10)	15 (8)	0.68 (0.24-1.9)	1.9 (0.5-7)

Monthly income (Ethiopian birr)				
<600 ETB	25 (42)	59 (33)	1.4 (0.8-2.6)	0.9 (0.47-2)
≥600 ETB	35 (58)	121 (67)	1	

Antenatal care (ANC)				
Yes	35 (58)	145 (81)	0.33 (0.18-0.63)	0.41 (0.18-0.93)^∗∗^
No	25 (42)	35 (19)	1	1

Hypertension disorder of pregnancy				
Yes	16 (27)	20 (11)	2.9 (1.3-6)	5 (1.9-13)^∗∗^
No	44 (73)	160 (89)	1	1

Urinary tract infection				
Yes	11 (18.3)	13 (7.2)	2.8 (1.2-6.8)	3.6 (1.1-11)^∗∗^
No	49 (81.7)	167 (92.8)	1	1

HIV/AIDS serostatus				
Positives	9 (15)	5 (2.7)	6.1 (1.98-19.2)	4.2 (0.9-18)^∗∗^
Not positive	51 (85)	175 (97.3)	1	1

History of preterm birth				
Yes	11 (18)	8 (4)	4.8 (1.8-12)	5 (1.6-15)^∗∗^
No	49 (82)	172 (96)	1	1

History of abortion				
Yes	23 (38)	42 (23.3)	2 (1.09-3.8)	2.3 (1.1-5)^∗∗^
No	37 (62)	138 (76.7)	1	1

MUAC (cm)				
<23	34 (57)	69 (38)	1	1
23-35	23 (38.)	84 (47)	0.55 (0.3-1.03)	0.54 (0.25-1.1)
>25	3 (5)	27 (15)	0.22 (0.064-0.79)	0.2 (0.046-0.96)^∗∗^

Note: ^∗^farmer, daily laborer, and student; ^∗∗^statistical significance *P* < 0.05.

## Data Availability

All the data included in the manuscript can be accessed from the corresponding author upon request via metanmann@gmail.com.
